# MRI-based lumbar cross-sectional indentation value is associated with spinopelvic sagittal balance in patients with degenerative kyphosis

**DOI:** 10.3389/fnins.2026.1896235

**Published:** 2026-09-17

**Authors:** Hongzhen Li, Shuai Xu, Chen Guo, Ke Ma, Haiying Liu, Yan Liang

**Affiliations:** 1 Department of Spine Surgery, Peking University People’s Hospital, Beijing, China; 2 Department of Orthopaedics, Huailai County Hospital, Zhangjiakou, Hebei, China

**Keywords:** degenerative kyphosis, lumbar cross-sectional indentation value, paraspinal muscle, pelvic tilt, PI-LL mismatch, sagittal balance, spinopelvic alignment

## Abstract

**Objective:**

To investigate whether MRI-based paraspinal muscle morphology, assessed using the lumbar cross-sectional indentation value (LCIV), is associated with spinopelvic sagittal balance in patients with degenerative kyphosis (DK).

**Methods:**

This retrospective case-control study included 81 patients with DK, defined as degenerative thoracolumbar kyphosis and/or loss of lumbar lordosis, and 60 surgical controls with symptomatic lumbar spinal stenosis but without degenerative spinal deformity. LCIV was measured from T12-L1 to L4-L5 on axial T2-weighted MRI, and the mean LCIV (mLCIV) was calculated. Spinopelvic sagittal balance in the DK group was evaluated using pelvic incidence minus lumbar lordosis (PI-LL) mismatch and pelvic tilt (PT). Associations among mLCIV, body mass index (BMI), thoracolumbar kyphosis, lumbar lordosis, and PT were also analysed.

**Results:**

Patients with DK had a lower mLCIV than controls (7.2 ± 5.4 mm vs. 10.8 ± 3.5 mm, *P* < 0.001). In the DK group, mLCIV was positively correlated with lumbar lordosis and BMI, and lumbar lordosis showed the stronger association in multivariate analysis. Patients with preserved PI-LL matching had a greater mLCIV than those with PI-LL mismatch (9.4 ± 5.2 mm vs. 5.8 ± 5.0 mm, *P* = 0.005). Similarly, patients with lower PT had a greater mLCIV than those with increased PT (8.5 ± 5.0 mm vs. 5.7 ± 5.4 mm, *P* = 0.028).

**Conclusion:**

Lower mLCIV was observed in patients with PI-LL mismatch and increased PT, suggesting that lower LCIV may accompany spinopelvic sagittal imbalance and compensatory pelvic retroversion in DK. LCIV may provide a simple MRI-based measure of posterior paraspinal muscle contour in sagittal balance evaluation.

## 1 Introduction

Degenerative kyphosis (DK) is a common sagittal deformity in middle-aged and older adults and is mainly associated with degenerative changes of the intervertebral discs and facet joints (Goh et al., 2017; Wang et al., 2019). Radiographically, DK is often characterized by local or global sagittal malalignment, including decreased lumbar lordosis (LL), increased thoracolumbar kyphosis (TLK), or both. Age, sex, and obesity have been reported to be associated with spinal degeneration and related musculoskeletal disorders (Fehlings et al., 2015; Hicks et al., 2005). Although obesity may accelerate disc degeneration and contribute to sagittal plane deformity (Kim et al., 2010), the development and progression of degenerative sagittal deformity cannot be explained by body mass index (BMI) alone. Recent studies have suggested that paraspinal muscle morphology may also play an important role in maintaining sagittal alignment and influencing the progression of spinal deformity (Kim et al., 2021; Roussouly and Pinheiro-Franco, 2011).

The paraspinal muscles are important active stabilizers of the spine and are closely involved in the maintenance of sagittal alignment and balance (Banno et al., 2017; Yagi et al., 2016). These muscles mainly include the psoas major, erector spinae, and multifidus muscles, and decreased paraspinal muscle strength or degeneration has been associated with low back pain and degenerative spinal deformity (Banno et al., 2017; Kalichman et al., 2010; Yagi et al., 2016). Muscle cross-sectional area (CSA) and fat infiltration are widely used to quantify paraspinal muscle degeneration and to evaluate its relationship with sagittal parameters (Guo et al., 2023a; Tian et al., 2024; Zhou et al., 2023). However, these measurements are relatively time-consuming and may be inconvenient for rapid clinical assessment. Takayama et al. (2016) proposed the lumbar cross-sectional indentation value (LCIV) as a simplified MRI-based parameter and showed that LCIV was correlated with conventional measurements of paraspinal muscle morphology, particularly in individuals older than 50 years. Previous LCIV-based work from our group described altered LCIV distribution in degenerative thoracolumbar kyphosis and its associations with local sagittal parameters (Guo et al., 2023b). However, whether LCIV differs according to PI-LL/PT-based sagittal balance status in DK remains insufficiently understood.

The SRS-Schwab classification uses pelvic incidence minus lumbar lordosis (PI-LL) and pelvic tilt (PT) to evaluate spinopelvic mismatch and compensatory pelvic retroversion in adult spinal deformity (Schwab et al., 2012). However, the relationship between LCIV-measured paraspinal muscle morphology and PI-LL/PT-based sagittal balance status in DK has not been fully clarified. We hypothesized that reduced paraspinal muscle morphology, as reflected by LCIV, may be associated with sagittal imbalance and compensatory pelvic retroversion in patients with DK. Therefore, this study aimed to investigate the association between LCIV and PI-LL/PT-based sagittal balance status in patients with DK. We further explored the relationships among LCIV, BMI, TLK, and LL to determine whether LCIV is more closely related to sagittal alignment than to body habitus alone.

## 2 Materials and methods

### 2.1 Study population and diagnostic criteria

This single-center retrospective case-control study was conducted between January 2016 and December 2019. Patients with degenerative thoracolumbar kyphosis (DTLK), loss of lumbar lordosis (LLL), or both were assigned to the degenerative kyphosis (DK) group. Participants who underwent surgery for symptomatic lumbar spinal stenosis but had no degenerative spinal deformity were assigned to the surgical control group. The study was approved by the Ethics Committee of Peking University People’s Hospital (approval No. 2022PHB417-001). The requirement for written informed consent was waived because of the retrospective design and the use of anonymised clinical data.

Based on previously reported small-to-moderate associations in the relevant literature, an expected correlation coefficient of |ρ| = 0.35 was selected for the primary association analysis. Using G*Power version 3.1 (Exact test family; Correlation: Bivariate normal model, two-tailed), with a two-sided α of 0.05 and a power of 0.90, at least 80 evaluable patients were required. Allowing for an anticipated 10% exclusion rate, 90 patients were targeted for the DK group. Accordingly, 90 patients with DK and 67 control participants were screened.

The inclusion criteria were as follows: (1) diagnosis of DK without coronal deformity for the DK group; (2) absence of degenerative spinal deformity for the control group; (3) availability of complete preoperative standing spine radiographs and lumbar MRI; (4) age > 50 years; and (5) SVA ≤ 9.5 cm according to the SRS-Schwab sagittal modifier (Schwab et al., 2012). Patients with SVA > 9.5 cm were excluded to limit the influence of marked global malalignment and associated whole-body compensation on the relationships under investigation.

The exclusion criteria were as follows: (1) coronal deformity; (2) incomplete or blurred radiographs; (3) adolescent idiopathic, congenital, or traumatic spinal deformity; (4) spinal tumor, infection, or spondylolisthesis; (5) previous lumbar spine surgery; (6) SVA > 9.5 cm; and (7) systemic disorders such as frailty syndrome (Davinelli et al., 2021).

Scoliosis was defined as a coronal Cobb angle > 10°. Coronal balance was defined as the distance between the central sacral vertical line and the midpoint of C7 < 30 mm.

### 2.2 MRI

All participants underwent MRI in the supine position using a 1.5-T Gyroscan ACS-NT scanner (Philips Medical Systems, Best, The Netherlands; matrix size, 512 × 512; field of view, 180 mm × 180 mm). T2-weighted images from T12-L1 to L4-L5 were obtained with a repetition time of 3,200 ms and an echo time of 102 ms. Sagittal and axial images were uploaded to the PACS workstation for subsequent measurements.

### 2.3 Measurement of parameters

Body mass index (BMI) was calculated as weight in kilograms divided by height in meters squared. Participants were categorized into normal-weight (N), overweight (OW), and obese (OB) groups according to World Health Organization criteria: 18.5 ≤ N < 25.0 kg/m^2^, 25.0 ≤ OW < 30.0 kg/m^2^, and OB ≥ 30.0 kg/m^2^.

Thoracolumbar kyphosis (TLK) was measured as the angle between the superior endplate of T10 and the inferior endplate of L2, and DTLK was defined as degenerative TLK ≥ 15°. LL was measured as the angle between the superior endplate of L1 and the superior endplate of S1, and LLL was defined as LL < 25°(Takayama et al., 2016). PI-LL mismatch and PT were used to evaluate sagittal malalignment and compensatory pelvic retroversion. PI was defined as the angle between a line perpendicular to the sacral endplate at its midpoint and a line connecting this point to the bicoxofemoral axis. PT was defined as the angle between the vertical plumb line and the line connecting the center of the bicoxofemoral axis to the midpoint of the superior endplate of S1 (Figure 1).

**FIGURE 1:**
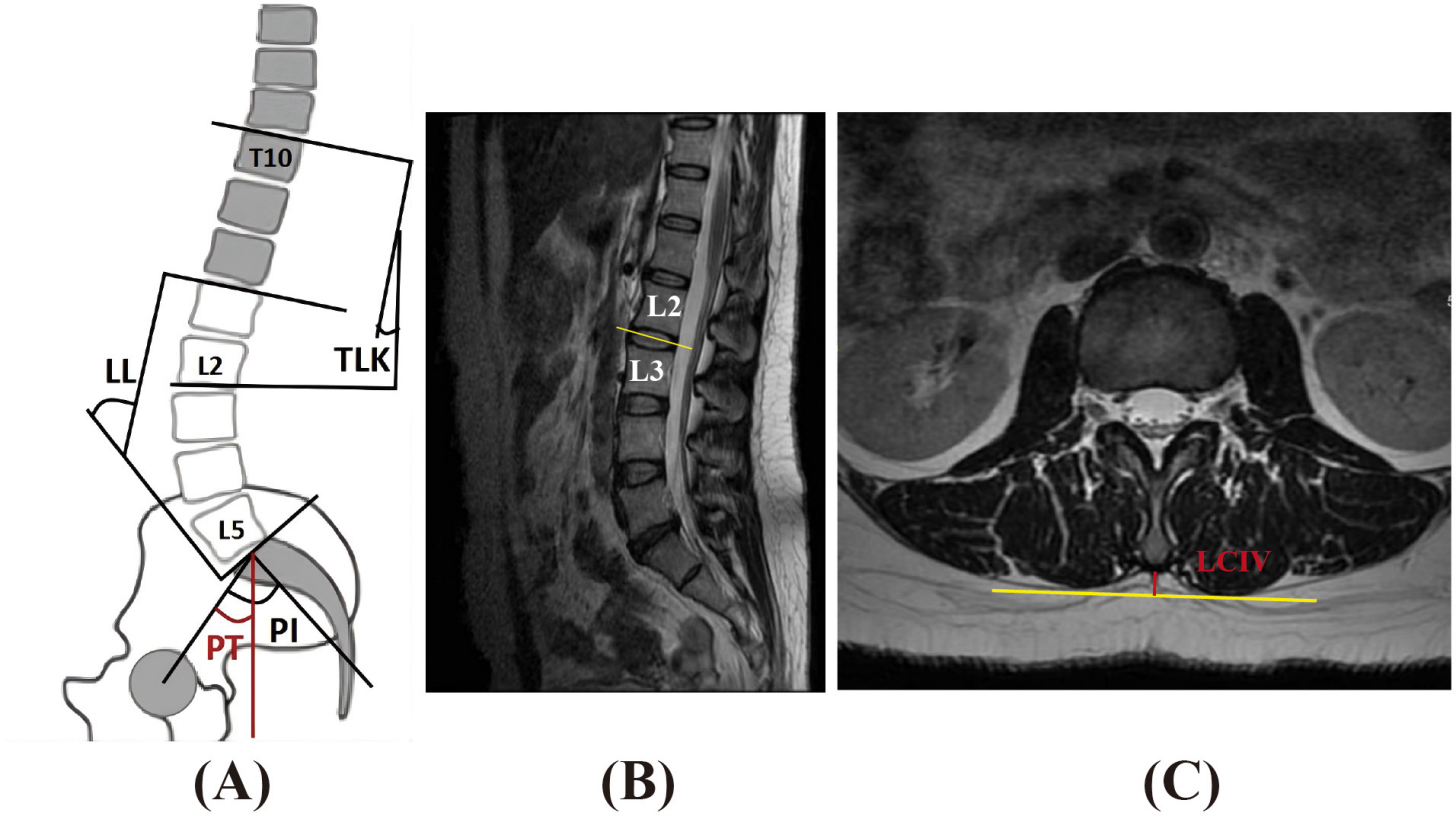
Radiographic and MRI-based measurement methods. **(A)** Definitions of TLK, LL, PI, and PT on a standing lateral radiograph. **(B)** Sagittal T2-weighted MRI for localization of the L2-L3 level. **(C)** Measurement of LCIV on axial T2-weighted MRI at the L2-L3 level. PT was defined as the angle between the vertical plumb line and the line connecting the center of the femoral head to the midpoint of the superior endplate of S1. PI was defined as the angle between a line perpendicular to the sacral endplate at its midpoint and a line connecting this point to the bicoxofemoral axis. LL, lumbar lordosis; TLK, thoracolumbar kyphosis; PI, pelvic incidence; PT, pelvic tilt; LCIV, lumbar cross-sectional indentation value; MRI, magnetic resonance imaging.

LCIV was defined as the perpendicular distance from the posterior tip of the spinous process to a reference line connecting the most posterior points of the bilateral paraspinal muscle contours on axial T2-weighted MRI (Figure 1). LCIV was measured separately at each level from T12-L1 to L4-L5. The L5-S1 level was excluded because the iliac crest and distinct muscular anatomy at this level may affect axial image acquisition and LCIV measurement. The mean LCIV (mLCIV) was calculated as the average LCIV from T12-L1 to L4-L5.

All parameters were independently measured by two reviewers. Reliability was assessed in all 141 participants, with repeat measurements after 2 weeks; reviewers were blinded to group allocation and clinical data. Interobserver and intraobserver reliability were assessed using two-way random- and mixed-effects ICC models, respectively, with absolute agreement and single measurements [ICC(2,1) and ICC(3,1), respectively]. ICC values were interpreted as follows: < 0.40, poor reliability; 0.40–0.75, fair to good reliability; and > 0.75, excellent reliability.

### 2.4 Subgroup analysis

Secondary subgroup analyses were conducted according to sex and BMI category (N, OW, and OB). The primary subgroup analysis focused on PI-LL and PT-based sagittal balance status in the DK group. According to PI-LL mismatch, patients were divided into the sagittal balance subgroup (SB-1, PI-LL ≤ 10°) and the sagittal imbalance subgroup (SIB-1, PI-LL > 10°). According to PT, patients were divided into the sagittal balance subgroup (SB-2, PT ≤ 20°) and the sagittal imbalance subgroup (SIB-2, PT > 20°).

### 2.5 Statistical analysis

Categorical variables were compared using the χ^2^ test or Fisher’s exact test, as appropriate. Normality and homogeneity of variance were assessed before group comparisons. Independent-samples *t*-tests or analysis of variance were used when parametric assumptions were met, and Mann–Whitney U or Kruskal–Wallis tests were used otherwise. Pearson correlation was used after consideration of linearity and influential observations. Variables were selected for multivariable linear regression based on clinical relevance and the preceding association analyses to identify factors associated with mLCIV and PT. Multicollinearity was assessed using variance inflation factors. Holm correction was applied to repeated level-specific LCIV comparisons. All statistical analyses were performed using SPSS software (version 22.0; IBM, Armonk, NY, USA). A two-sided *P*-value < 0.05 was considered statistically significant.

## 3 Results

### 3.1 Baseline characteristics and measurement reliability

A total of 81 patients with DK and 60 control participants were included in the final analysis after exclusion of nine and seven initially screened participants, respectively, because of incomplete preoperative radiographic and/or MRI data. Among the patients with DK, 55 had DTLK alone, 23 had both DTLK and LLL, and 3 had isolated LLL. The two groups were comparable in sex distribution, age, and BMI, whereas TLK was greater and LL was lower in the DK group than in the control group (Table 1). Intraobserver reproducibility and interobserver reliability for LCIV and sagittal parameters showed good-to-excellent agreement, supporting the reliability of the radiographic and MRI-based measurements used in this study (Table 2).

**TABLE 1:** Baseline characteristics of the DK and surgical control groups.

Characteristic	DK group	Control group	*P*
Sex			0.835
Male	27	19	
Female	54	41	
Age, y	66.1 ± 8.9	66.3 ± 9.7	0.891
BMI, kg/m^2^	26.0 ± 3.4	26.2 ± 3.9	0.741
18.5–24.9	34	25	
25–29.9	32	26	
≥ 30	15	9	< 0.001
TLK, °	22.0 ± 14.4	7.5 ± 4.3	
LL, °	34.4 ± 20.7	42.4 ± 6.6	0.002

DK, degenerative kyphosis; BMI, body mass index; TLK, thoracolumbar kyphosis; LL, lumbar lordosis.

**TABLE 2:** Intraobserver reproducibility and interobserver reliability for all parameters.

Parameter	Intraobserver ICC (95% CI)	Interobserver ICC (95% CI)
LCIV T12-L1, mm	0.83 (0.77–0.88)	0.76 (0.68–0.81)
LCIV L1-L2, mm	0.72 (0.63–0.79)	0.70 (0.61–0.78)
LCIV L2-L3, mm	0.89 (0.87–0.92)	0.81 (0.73–0.86)
LCIV L3-L4, mm	0.92 (0.89–0.94)	0.77 (0.69–0.83)
LCIV L4-L5, mm	0.88 (0.84–0.91)	0.81 (0.75–0.87)
TLK, °	0.86 (0.81–0.90)	0.76 (0.66–0.82)
LL, °	0.81 (0.73–0.85)	0.75 (0.67–0.81)
PI, °	0.89 (0.85–0.92)	0.79 (0.72–0.85)
PT, °	0.78 (0.70–0.84)	0.79 (0.73–0.86)

ICC, intraclass correlation coefficient; CI, confidence interval; LCIV, lumbar cross-sectional indentation value; TLK, thoracolumbar kyphosis; LL, lumbar lordosis; PI, pelvic incidence; PT, pelvic tilt.

### 3.2 Descriptive comparison of LCIV between the DK and control groups

As a descriptive comparison, the DK group showed a lower mLCIV than the control group (7.2 ± 5.4 mm vs. 10.8 ± 3.5 mm, *P* < 0.001). After Holm correction, segmental LCIV remained significantly lower in the DK group from T12-L1 to L2-L3, whereas the differences at L3-L4 and L4-L5 were not significant (Table 3). Within the DK group, LCIV showed an increasing trend from T12-L1 to L4-L5 (*P* < 0.001), suggesting that paraspinal muscle morphology was more affected at the thoracolumbar and upper lumbar levels.

**TABLE 3:** Comparison of LCIV between the DK and control groups.

LCIV, mm	DK group	Control group	*P*
T12-L1	4.4 ± 5.3	9.7 ± 4.2	< 0.001
L1-L2	5.0 ± 5.8	10.0 ± 4.2	< 0.001
L2-L3	6.0 ± 5.9	10.1 ± 4.2	< 0.001
L3-L4	8.1 ± 6.7	10.4 ± 4.2	0.074
L4-L5	12.4 ± 6.7	13.8 ± 4.1	0.190
mLCIV	7.2 ± 5.4	10.8 ± 3.5	< 0.001

*P*-values for the five level-specific LCIV comparisons were adjusted using the Holm method; mLCIV was analyzed separately. LCIV, lumbar cross-sectional indentation value; DK, degenerative kyphosis.

Sex- and BMI-based subgroup analyses were performed as secondary descriptive analyses. In the control group, females had a lower mLCIV than males (10.0 ± 3.0 mm vs. 12.8 ± 4.1 mm, *P* = 0.007), whereas no significant sex-related difference in mLCIV was observed in the DK group. In the control group, mLCIV differed among the normal-weight, overweight, and obese subgroups (*P* = 0.030), mainly at the T12-L1 and L1-L2 levels between the normal-weight and obese subgroups (Figure 2).

**FIGURE 2:**
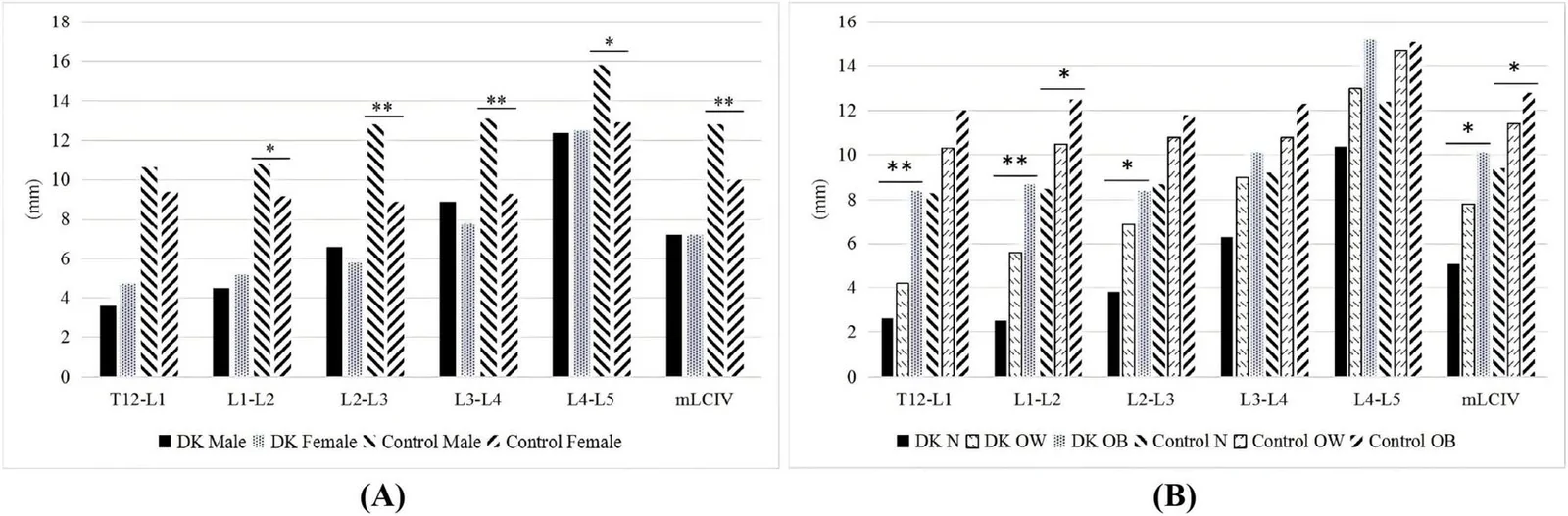
Secondary subgroup analyses of LCIV according to sex and BMI. **(A)** Comparisons of LCIV between males and females in the DK and control groups. **(B)** Comparisons of LCIV among the normal-weight, overweight, and obese subgroups in the DK and control groups. *P < 0.05; **P < 0.01. LCIV, lumbar cross-sectional indentation value; DK, degenerative kyphosis; BMI, body mass index; N, normal weight; OW, overweight; OB, obese.

### 3.3 Associations among LCIV, BMI, and sagittal alignment parameters

In the control group, mLCIV was positively correlated with BMI and negatively correlated with TLK. In the DK group, mLCIV was positively correlated with both BMI and LL, but not with TLK (Table 4). When all participants were analysed together, mLCIV was positively correlated with BMI and LL and negatively correlated with TLK. In multivariate regression analysis, BMI and LL were independently associated with mLCIV in the DK group, with LL showing the stronger standardized effect. In the control group, BMI and TLK were independently associated with mLCIV. In the overall cohort, BMI, TLK, and LL were all associated with mLCIV (Table 5). No substantial multicollinearity was identified, with all VIF values < 5. Together, these results indicate that mLCIV was influenced by body habitus and was more strongly related to LL in patients with DK.

**TABLE 4:** Correlation analysis among BMI, TLK, LL, and mLCIV.

Group	Variable	BMI		TLK		LL	
		r	*P*	r	*P*	r	*P*
DK group	TLK	0.116	0.304				
	LL	0.039	0.727	0.315	0.004		
	mLCIV	0.290	0.009	−0.062	0.612	0.603	< 0.001
Control group	TLK	−0.034	0.794				
	LL	0.196	0.133	0.034	0.794		
	mLCIV	0.412	0.002	−0.304	0.018	0.093	0.510
All cases	TLK	0.046	0.591				
	LL	0.069	0.420	0.114	0.179		
	mLCIV	0.297	0.001	−0.271	0.003	0.544	< 0.001

BMI, body mass index; TLK, thoracolumbar kyphosis; LL, lumbar lordosis; LCIV, lumbar cross-sectional indentation value; DK, degenerative kyphosis; r, correlation coefficient.

**TABLE 5:** Multiple linear regression analyses for mLCIV in the DK group, control group, and overall cohort.

Group	Variable	B	SE	95% CI	Beta	t	*P*
DK group	Constant	−10.403	3.899	−18.165, −2.641		−2.668	0.010
	BMI	0.459	0.144	0.172, 0.746	0.292	3.192	0.002
	LL	0.156	0.024	0.108, 0.204	0.603	6.600	< 0.001
Control group	Constant	3.551	3.012	−2.480, 9.582		1.179	0.244
	BMI	0.348	0.108	0.132, 0.564	0.398	3.218	0.002
	TLK	−0.243	0.105	−0.453, −0.033	−0.285	−2.306	0.025
All cases	Constant	−6.589	2.434	−11.402, −1.776		−2.707	0.008
	BMI	0.413	0.088	0.239, 0.587	0.305	4.683	< 0.001
	TLK	−0.133	0.024	−0.180, −0.086	−0.362	−5.518	< 0.001
	LL	0.174	0.020	0.134, 0.214	0.581	8.874	< 0.001

Model fit: DK group, R^2^ = 0.448, adjusted R^2^ = 0.434; control group, R^2^ = 0.251, adjusted R^2^ = 0.224; overall cohort, R^2^ = 0.505, adjusted R^2^ = 0.494. LCIV, lumbar cross-sectional indentation value; DK, degenerative kyphosis; BMI, body mass index; LL, lumbar lordosis; TLK, thoracolumbar kyphosis; SE, standard error; CI, confidence interval.

### 3.4 LCIV according to PI-LL and PT-based sagittal balance status

#### 3.4.1 LCIV difference between the PI-LL balance and imbalance subgroups

According to PI-LL mismatch, 31 patients were classified into the SB-1 subgroup and 50 into the SIB-1 subgroup. Sex distribution, age, and BMI were comparable between the two subgroups, suggesting that the observed differences in LCIV were unlikely to be primarily explained by demographic characteristics or body habitus. TLK did not differ significantly between the subgroups (25.0 ± 13.2° vs. 20.2 ± 14.9°, *P* = 0.141), whereas LL was greater in the SB-1 subgroup than in the SIB-1 subgroup (47.2 ± 12.8° vs. 26.5 ± 20.8°, *P* < 0.001).

LCIV was greater in the SB-1 subgroup than in the SIB-1 subgroup, with significant differences mainly observed from L2-L3 to L4-L5. The mLCIV was also significantly higher in the SB-1 subgroup than in the SIB-1 subgroup (9.4 ± 5.2 mm vs. 5.8 ± 5.0 mm, *P* = 0.005) (Table 6). These findings indicate that patients with preserved PI-LL matching had greater mLCIV than those with PI-LL mismatch.

**TABLE 6:** Comparisons of LCIV in terms of PI-LL and PT.

LCIV, mm	PI–LL				PT			
	SB-1 (*n* = 31)	SIB-1 (*n* = 50)	*P*	Cohen’s d (95% CI)	SB-2 (*n* = 44)	SIB-2 (*n* = 37)	*P*	Cohen’s d (95% CI)
T12–L1	5.2 ± 6.1	3.9 ± 4.6	0.279	0.25 (−0.20, 0.70)	5.2 ± 5.3	3.5 ± 5.1	0.268	0.33 (−0.11, 0.77)
L1–L2	6.6 ± 6.1	4.0 ± 5.5	0.102	0.45 (0.00, 0.91)	6.0 ± 6.0	3.8 ± 5.4	0.268	0.38 (−0.06, 0.82)
L2–L3	8.2 ± 6.1	4.6 ± 5.4	0.021	0.63 (0.18, 1.09)	7.0 ± 5.8	4.9 ± 5.9	0.268	0.36 (−0.08, 0.80)
L3–L4	11.6 ± 6.4	5.9 ± 6.0	< 0.001	0.93 (0.46, 1.40)	10.0 ± 6.2	6.0 ± 6.8	0.033	0.62 (0.17, 1.06)
L4–L5	15.5 ± 5.4	10.5 ± 6.8	0.003	0.79 (0.33, 1.26)	14.3 ± 5.9	10.3 ± 7.0	0.033	0.62 (0.17, 1.07)
mLCIV	9.4 ± 5.2	5.8 ± 5.0	0.005	0.71 (0.25, 1.17)	8.5 ± 5.0	5.7 ± 5.4	0.028	0.54 (0.09, 0.99)

*P*-values for level-specific LCIV comparisons were adjusted using the Holm method; mLCIV was analysed separately. LCIV, lumbar cross-sectional indentation value; mLCIV, mean lumbar cross-sectional indentation value; PI, pelvic incidence; LL, lumbar lordosis; PT, pelvic tilt; SB, sagittal balance; SIB, sagittal imbalance; CI, confidence interval.

#### 3.4.2 LCIV difference between the PT balance and imbalance subgroups

According to PT, 44 patients were classified into the SB-2 subgroup and 37 into the SIB-2 subgroup. Sex distribution, age, and BMI were not significantly different between the two subgroups. LL was greater in the SB-2 subgroup than in the SIB-2 subgroup (39.2 ± 19.8° vs. 28.8 ± 20.5°, *P* = 0.022), indicating that increased pelvic retroversion was accompanied by reduced lumbar lordosis.

The SB-2 subgroup showed greater LCIV at L3-L4 and L4-L5 than the SIB-2 subgroup, and mLCIV was also significantly higher in the SB-2 subgroup (8.5 ± 5.0 mm vs. 5.7 ± 5.4 mm, *P* = 0.028) (Table 6). PT was negatively correlated with both LL (*r* = −0.351, *P* = 0.001) and mLCIV (r = −0.271, *P* = 0.024). In multivariate regression analysis, LL was independently associated with PT in the DK group (β = −0.397, *P* = 0.006). These findings suggest that lower LCIV may coexist with increased pelvic retroversion, although LL remained the dominant radiographic factor associated with PT (Figure 3).

**FIGURE 3:**
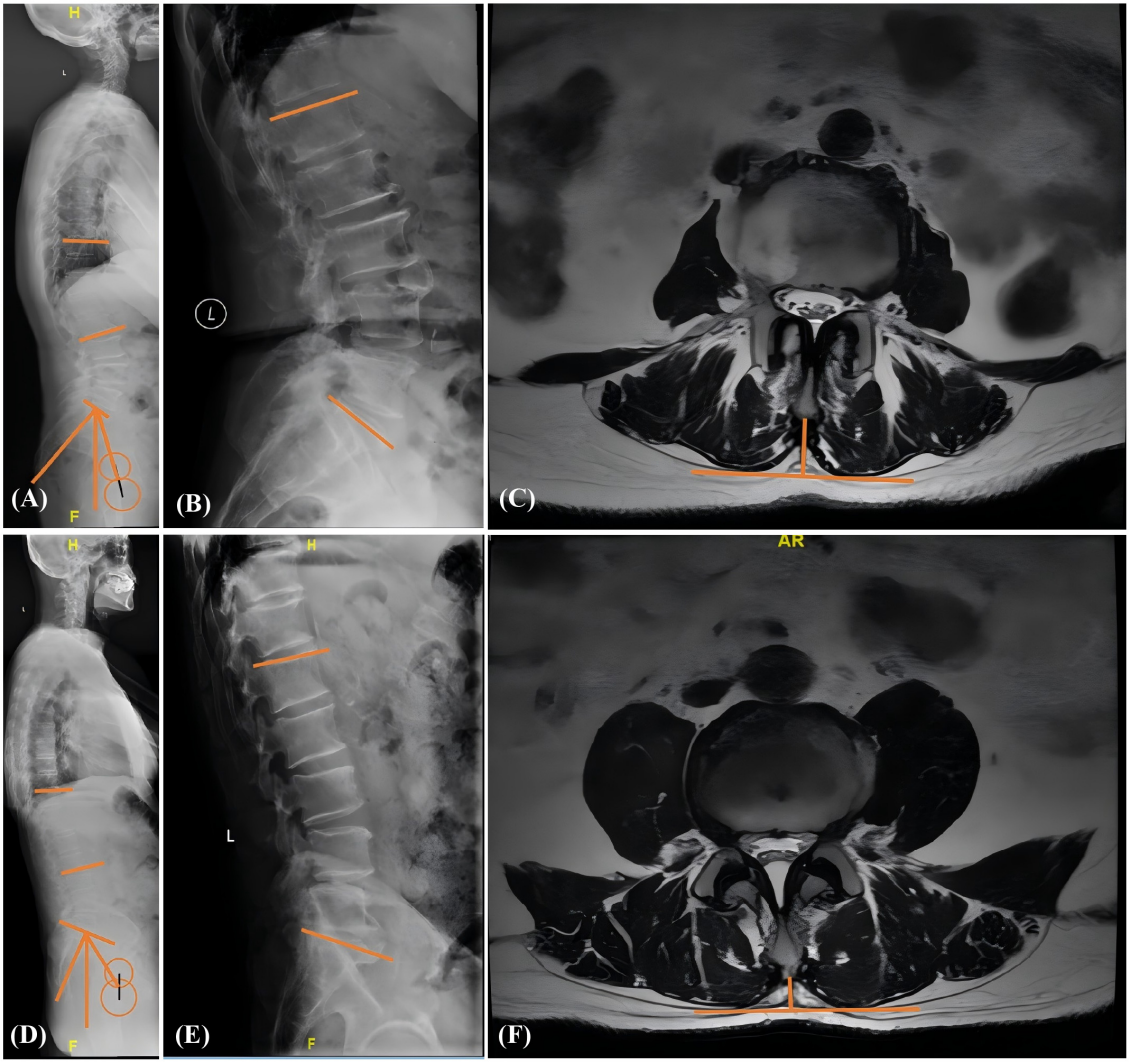
Representative cases illustrating different PI-LL and PT-based sagittal balance status in patients with DK. **(A–C)** A 68-year-old female with preserved sagittal balance: BMI, 23.7 kg/m^2^; PI-LL, 1.0°; PT, 9.2°; LL, 40.4°; TLK, 22.8°; LCIV at L2-L3, 13.6 mm; and mLCIV, 11.6 mm. **(D–F)** A 70-year-old female with sagittal imbalance and increased pelvic retroversion: BMI, 24.7 kg/m^2^; PI-LL, 21.1°; PT, 30.3°; LL, 24.2°; TLK, 15.2°; LCIV at L2-L3, 7.4 mm; and mLCIV, 7.7 mm. DK, degenerative kyphosis; LL, lumbar lordosis; TLK, thoracolumbar kyphosis; PI, pelvic incidence; PT, pelvic tilt; LCIV, lumbar cross-sectional indentation value; BMI, body mass index; mLCIV, mean lumbar cross-sectional indentation value.

## 4 Discussion

The spinal stability system is generally considered to involve passive spinal structures, active muscular support, and neuromuscular control (Goh et al., 2017; Yagi et al., 2016). In degenerative sagittal deformity, changes in the intervertebral discs, facet joints, ligaments, and paraspinal muscles may jointly affect the maintenance of sagittal alignment (Banno et al., 2017; Tian et al., 2024; Wang et al., 2019; Yagi et al., 2016). DK is characterized by decreased LL, increased TLK, or both, and its progression may be influenced not only by skeletal degeneration but also by body habitus and paraspinal muscle morphology (Kalichman et al., 2010; Roussouly and Pinheiro-Franco, 2011). Building on this rationale, the present study focused on PI-LL/PT-based sagittal balance status in patients with DK and found that patients with PI-LL mismatch or increased PT had lower mLCIV than those with preserved PI-LL matching or lower PT. These findings suggest that reduced MRI-based paraspinal muscle morphology may accompany spinopelvic sagittal imbalance and compensatory pelvic retroversion.

Various methods are available to assess the paraspinal muscles, including muscle cross-sectional area and fat infiltration, but these methods are relatively complex and time-consuming in routine clinical practice (Kokosova et al., 2025; Ranger et al., 2017). Takayama et al. (2016) proposed LCIV as a simplified imaging-based parameter and reported its correlation with conventional assessments of paraspinal muscle morphology. Previous studies have suggested that paraspinal muscle degeneration and reduced posterior extensor function may be related to LL loss and degenerative sagittal deformity (Nagata et al., 2026; Shafaq et al., 2012). In the present study, LCIV was used as a practical measure of posterior paraspinal muscle contour rather than as a replacement for detailed quantitative muscle evaluation. Therefore, LCIV should be interpreted as a simple morphological indicator and not as direct evidence of paraspinal muscle degeneration or as a direct measurement of muscle composition, fat infiltration, functional cross-sectional area, or strength.

The relationship between body habitus and sagittal imbalance remains complex. Obesity and altered body composition have been associated with spinal degeneration, low back symptoms, and degenerative deformity (Hicks et al., 2005; Shiri et al., 2013). Wang et al. (2019) reported that BMI was closely associated with the development and progression of degenerative scoliosis, whereas other studies have suggested that sagittal sequence loss may be more closely related to absolute muscle loss or muscle degeneration than to BMI alone (Fu et al., 2014; Goh et al., 2017). Kim et al. (2010) also reported that BMI was not necessarily correlated with sagittal imbalance in degenerative kyphosis. Consistent with this concept, the present study found that BMI was associated with mLCIV, but BMI was not the primary determinant of sagittal balance status in patients with DK. Instead, LL showed a stronger association with mLCIV in the DK group and was also associated with PT.

Previous studies have shown that different paraspinal muscle groups may contribute differently to lumbar curvature, pelvic parameters, and global sagittal alignment (Banno et al., 2017; Yagi et al., 2016; Zhou et al., 2023). The interaction between spinal deformity and paraspinal muscle degeneration may also be understood within the broader concept of mechanically driven deformity progression, in which altered loading and muscular imbalance may reinforce deformity development (Mannion et al., 1998). Therefore, LCIV in patients with DK should be interpreted in the context of sagittal alignment rather than as a simple reflection of body size.

In this study, LCIV was lower in the DK group than in the control group, with significant differences from T12-L1 to L2-L3 after Holm correction, whereas the differences at L3-L4 and L4-L5 were not significant. This segmental pattern may be related to the anatomical distribution of degenerative kyphosis, which often involves the thoracolumbar junction and upper lumbar spine. In contrast, the lower lumbar paraspinal region is influenced by the iliac crest and sacral attachments, which may partly explain the relatively preserved LCIV at L4-L5 (Ghiasi et al., 2016; Takayama et al., 2016). In addition, LCIV was strongly correlated with LL in the DK group, indicating a close association between LCIV-defined paraspinal muscle morphology and lumbar lordosis. Hongo et al. (2012) reported that decreased back extensor strength was associated with reduced LL and kyphotic alignment in older adults, which is consistent with the relationship between reduced LCIV and LL loss observed in the present study. However, because this study was cross-sectional, whether reduced LCIV contributes to LL loss or develops secondary to sagittal deformity cannot be determined.

In degenerative sagittal deformity, sagittal correction and LL restoration are clinically important because postoperative sagittal malalignment may be associated with mechanical complications such as proximal junctional kyphosis (Pan et al., 2024). The SRS-Schwab classification provides widely used radiographic thresholds for evaluating sagittal deformity, including PI-LL mismatch and PT (Slattery and Verma, 2018). Therefore, in the present study, patients with DK were divided into balance and imbalance subgroups according to PI-LL and PT. Using these thresholds, the present study showed a consistent reduction in mLCIV among patients classified as sagittally imbalanced by either PI-LL mismatch or increased PT. This result extends the relevance of LCIV from local kyphotic or lordotic parameters to PI-LL/PT-defined spinopelvic malalignment and pelvic compensation.

This relationship should be interpreted cautiously. PI-LL includes LL as a component, and LCIV was strongly associated with LL in the DK group. Therefore, the observed association between LCIV and PI-LL mismatch may partly reflect the close relationship among LCIV, LL, and spinopelvic matching. Similarly, although PT was negatively correlated with mLCIV, multivariate analysis indicated that LL remained the independent factor associated with PT. These findings suggest that LCIV may not directly determine pelvic compensation, but it may represent a clinically accessible marker of paraspinal morphology accompanying LL loss and sagittal imbalance.

From a clinical perspective, LCIV may provide an easily obtainable MRI-based indicator of paraspinal muscle morphology in patients with DK and may help contextualize spinopelvic imbalance during preoperative assessment. Its potential usefulness for surgical planning and rehabilitation warrants further investigation in studies incorporating clinical and functional outcomes (Massé-Alarie et al., 2017; Weber et al., 2018). LCIV should be interpreted together with established sagittal alignment parameters, including LL, PI-LL, and PT, as well as clinical symptoms and functional status.

This study has several limitations. First, LCIV is a simplified morphological parameter that does not directly assess muscle cross-sectional area, fat infiltration, or functional strength. As an absolute distance measurement, it may also be influenced by body size and skeletal dimensions. Marked fatty infiltration may further affect identification of the muscle contour. Second, this was a single-center retrospective cross-sectional study using surgical controls with symptomatic lumbar spinal stenosis, which may have influenced paraspinal muscle morphology and LCIV. Causal relationships therefore cannot be established, and larger prospective studies are needed. Third, because PI-LL includes LL as a component and LCIV was strongly associated with LL, the association between LCIV and PI-LL mismatch should be interpreted together with the relationship between LCIV and lumbar lordosis. These findings are most applicable to patients without marked global sagittal malalignment and may not generalize to other types of spinal deformity.

## 5 Conclusion

In patients with DK, lower mLCIV was observed in those with PI-LL mismatch or increased PT, suggesting that LCIV-defined paraspinal muscle morphology may accompany spinopelvic sagittal imbalance and compensatory pelvic retroversion. LCIV was also closely related to LL, indicating that this imaging feature may be associated with lumbar lordosis in DK. Although BMI influenced LCIV, it was not the primary focus of sagittal balance assessment. LCIV may serve as a simple MRI-based marker of posterior paraspinal muscle morphology in the context of spinopelvic sagittal balance, but further prospective studies incorporating quantitative muscle composition and clinical outcomes are needed.

## Data Availability

The original contributions presented in this study are included in this article/supplementary material, further inquiries can be directed to the corresponding author.
